# Shoulder and genetics: what orthopedic surgeons need to know

**DOI:** 10.1016/j.xrrt.2026.100730

**Published:** 2026-03-23

**Authors:** Ulas Can Kolac, Alp Paksoy, Dilsu Dicle Erkan, Doruk Akgun

**Affiliations:** a Hacettepe University Faculty of Medicine, Department of Orthopedics and Traumatology, Ankara, Turkey; b Charité University Hospital, Center for Musculoskeletal Surgery, Berlin, Germany; c Department of Medical Genetics, Ankara Etlik City Hospital, Ankara, Türkiye

**Keywords:** Genetics, Rotator cuff tears, Adhesive capsulitis, Glenohumeral osteoarthritis, Shoulder instability, Shoulder

## Abstract

Common shoulder disorders are a major source of pain and disability worldwide. Although mechanical overload, degeneration, and trauma are well-recognized contributors, substantial variability in disease onset, severity, and response to treatment suggests that biological and inherited factors may also influence disease susceptibility and progression. In recent years, advances in genomic and molecular research have provided new insights into the mechanisms underlying common shoulder pathologies.

Evidence from family-based studies, genome-wide association studies, transcriptomic analyses, and causal inference approaches indicates that several common shoulder conditions demonstrate a measurable genetic component. Rotator cuff disease and adhesive capsulitis show the strongest evidence for inherited susceptibility, involving pathways related to extracellular matrix remodeling, inflammation, and fibrosis. Glenohumeral osteoarthritis is primarily characterized by altered gene expression within diseased cartilage and periarticular tissues, although shoulder-specific inherited risk data remain limited. Shoulder instability has been associated with variation in genes involved in collagen synthesis and capsular remodeling, though the distinction between inherited predisposition and secondary molecular responses to injury remains incompletely defined.

These findings support the concept that common shoulder pathologies are multifactorial disorders influenced by both genetic susceptibility and disease-specific molecular processes. This narrative review aims to synthesize and contextualize current evidence on the role of genetics in 4 prevalent shoulder conditions: rotator cuff tears, adhesive capsulitis, glenohumeral osteoarthritis, and shoulder instability, with the goal of providing an accessible overview for practicing orthopedic surgeons.

Shoulder disorders are a major source of pain, disability, and the need for specialized orthopedic management.^32^^,^^34^ Conditions such as rotator cuff tears (RCTs), adhesive capsulitis, glenohumeral osteoarthritis (GHOA), and shoulder instability are some of the most frequently encountered pathologies in orthopedics.^14^^,^^22^^,^^49^^,^^50^ While mechanical and systemic risk factors such as overuse, degeneration, trauma, and metabolic comorbidities are well-documented, considerable variation persists in disease onset, severity, and response to treatment.^33^ This variability has led to increased attention toward genetics as a factor in shoulder pathologies.^17^^,^^41^^,^^42^

Recent advances in genomic science have changed the way we look at biological mechanisms of musculoskeletal conditions.^29^^,^^31^ Large-scale genome-wide association studies (GWASs), transcriptomic analyses of shoulder tissues, and causal inference approaches such as Mendelian randomization have shown us associations between shoulder pathologies and genetics, shown in Table I.^6^^,^^15^^,^^24^ These studies have identified specific risk loci and gene expression signatures associated with tendon degeneration, joint capsule fibrosis, cartilage breakdown, and connective tissue remodeling processes, which seem to play an important role in some shoulder pathologies.^15^^,^^24^^,^^42^^,^^44^

**Table I:** Key genetic concepts relevant to shoulder pathologies.

Term	Definition	Relevance to shoulder pathologies
Genome-wide association study (GWAS)	A method that scans the entire genome of many individuals to find genetic variants associated with specific diseases or traits.	GWASs have identified loci associated with rotator cuff tears, shoulder pain, and osteoarthritis.
Single nucleotide polymorphism (SNP)	A variation at a single position in the DNA sequence among individuals.	Commonly used in GWAS to locate genes associated with increased disease risk.
Transcriptomics	The study of RNA transcripts produced by the genome under specific circumstances or in a specific tissue.	Helps identify gene expression changes in shoulder tissues (eg, rotator cuff tendon, joint capsule).
Gene expression signature	A unique pattern of gene activity that reflects biological states or disease processes.	Distinct signatures have been associated with tendon degeneration and capsular fibrosis.
Mendelian randomization	A causal inference method that uses genetic variants as proxies for modifiable exposures (eg, obesity, lipid levels) to determine their effect on disease.	Used to infer causal links between metabolic risk factors and shoulder conditions like osteoarthritis or frozen shoulder.
Heritability	The proportion of variation in a trait that can be attributed to genetic differences.	High heritability has been reported in some shoulder conditions, supporting a strong genetic component.
Polygenic risk score (PRS)	A measure of an individual's genetic risk for a condition based on the cumulative effect of many genetic variants.	Not yet widely used in clinical orthopedics but could inform future risk stratification models.
Candidate gene study	Investigates prespecified genes thought to be associated with a disease based on known biology.	Early studies of rotator cuff tears examined collagen genes, though often limited by small sample sizes.
eQTL (expression Quantitative Trait Loci)	Genetic loci that explain variation in gene expression levels.	Help link genetic variants to downstream gene expression changes in shoulder tissues.
Epigenetics	Study of heritable changes in gene expression that do not involve alterations to the DNA sequence (eg, methylation).	Epigenetic modifications may play a role in tendon aging, fibrosis, and healing potential.

While there is some literature explaining the genetic basis of diseases like hip and knee osteoarthritis (OA), shoulder conditions have only recently come under similar genomic investigation.^19^^,^^47^ New evidence shows that common shoulder pathologies including RCTs, adhesive capsulitis, GHOA, and shoulder instability demonstrate genetic and molecular signatures. These range from familial aggregation and high-risk loci identified through GWAS to transcriptomic profiles indicating tendon degeneration, capsular fibrosis, or altered inflammatory signaling. As the literature continues to expand, understanding these genetic factors is becoming increasingly important for orthopedic surgeons, both to better explain clinical heterogeneity and to anticipate future applications in precision diagnostics and individualized treatment strategies.

In this narrative review, we provide a summary of the current genetic evidence involving 4 common shoulder pathologies (RCTs, adhesive capsulitis, GHOA, and shoulder instability). This review aims to give orthopedic surgeons a practical, up-to-date perspective on the role of genetics in shoulder pathologies and its implications for research and clinical care.

## Rotator cuff tears

RCTs are one of the most common degenerative musculoskeletal disorders encountered in clinical practice, particularly affecting individuals over the age of 50.^46^ It involves a large spectrum of pathologies ranging from subacromial impingement and tendinopathy to partial and full-thickness tendon tears. While mechanical overload, trauma, and age-related degeneration have been commonly considered as primary causes, new evidence has changed our understanding of its etiology.^33^^,^^42^ Recent studies have shown that genetic predisposition plays a substantial role in determining an individual's susceptibility to rotator cuff pathologies.^11^^,^^33^

Genetic evidence in RCTs derives from several complementary study designs. Family-based and genealogical studies assess clustering of disease within relatives and suggest inherited susceptibility. Candidate gene studies evaluate predefined genes involved in tendon biology, while GWAS examine genetic variation across the entire genome to identify unbiased risk loci. Transcriptomic analyses, in contrast, measure gene expression within diseased tendon tissue and primarily reflect active molecular changes rather than inherited predisposition.

Studies about the heritability of RCTs emerged from clinical observations and familial studies. Harvie et al demonstrated that siblings of individuals with full-thickness RCTs had over twice the risk of developing a similar tear and nearly 5 times the risk of developing symptoms compared to age-matched controls.^18^ Gwilym et al further extended this observation in a 5-year longitudinal study, showing that patients with a family history not only had an increased likelihood of developing a tear but also exhibited a higher rate of progression and symptom development over time.^16^ In a larger population-based genealogical analysis, Tashjian and colleagues confirmed that RCTs tend to aggregate in families, particularly in individuals with early-onset disease, further supporting a strong inherited component.^42^ Collectively, these family-based studies provide consistent evidence for inherited susceptibility, particularly in early-onset or progressive rotator cuff disease.

Building on early observations, several gene studies have investigated the molecular pathobiological basis of RCTs by examining variations in genes related to tendon biology, extracellular matrix (ECM) regulation, and inflammatory pathways.^33^ In a systematic review by Longo et al, associations were found between RCTs and polymorphisms in genes such as *DEFB1*, *FGFR1*, *FGFR3*, *ESRRB*, *FGF10*, and *FGF3*.^33^ Among these candidates, the *ESRRB* gene, encoding a nuclear hormone receptor crucial for cellular homeostasis, was repeatedly highlighted.^33^ Teerlink et al confirmed its significance by identifying 2 specific high-risk *ESRRB* haplotypes (with odds ratios of approximately 6.9 and 5.3) that were significantly more prevalent in individuals with full-thickness RCTs compared to genetically matched controls.^45^ Although candidate gene studies provide biologically plausible associations, their findings are hypothesis-driven and often require validation in larger population-based cohorts.

The first GWAS of RCTs identified 2 single-nucleotide polymorphisms located in the *SAP30BP* and *SASH1* genes.^43^ Both genes are involved in cell cycle regulation, apoptosis, and tissue homeostasis.^43^ While these findings were compelling, larger and more powered studies have since expanded the genomic landscape of rotator cuff disease.^24^ A more recent GWAS and meta-analysis conducted by Kim et al, involving over 21,000 patients from the UK Biobank and Kaiser Permanente cohorts, identified 11 genome-wide significant loci associated with either RCTs or shoulder impingement.^24^ These included novel variants in or near the genes *SLC39A8*, *UBE2D3*, *STK24*, *FRMPD4*, *ACOT9*, and *SAT1*.^24^ Several of these loci are involved in pathways regulating protein degradation, inflammation, and synaptic function, suggesting complex and diverse biological contributions to tendon disease.^24^

To support the functional relevance of these findings, Kim et al also performed transcriptomic analyses of torn versus intact rotator cuff tissue and found significant upregulation of *STK24*, downregulation of *SAT* and *UBE2D3* in torn tendons; linking genetic variants to changes in gene expression and tissue phenotype.^24^ GWAS and meta-analytic approaches currently represent the strongest level of genetic evidence in rotator cuff disease, as they assess large populations and reduce selection bias inherent to candidate gene studies.

Complementing these findings, Plachel et al demonstrated that patients with chronic rotator cuff tendinopathy and degenerative RCTs exhibit distinct circulating microRNA profiles.^39^ Several miRNAs were downregulated in both serum and tendon tissue, suggesting coordinated epigenetic regulation across systemic and local environments. These miRNA profiles show promise as minimally invasive diagnostic or prognostic biomarkers for degenerative rotator cuff pathology. Importantly, transcriptomic and microRNA studies characterize molecular activity within already diseased tissue and therefore reflect mechanisms of degeneration and healing rather than primary inherited risk.

Additionally, a transcriptomic study by Fan et al identified distinct gene expression patterns between diabetic and nondiabetic patients with RCTs, suggesting that diabetes-related changes in oxidative stress and ECM regulation may impair tendon healing; however, these findings require further validation.^12^ These data support the hypothesis that single-nucleotide polymorphisms involved in gene regulation may directly influence tendon vulnerability, healing capacity, and degenerative progression^24^ (Table II).

**Table II:** Summary of key genetic studies in rotator cuff tears.

Study	Study type	Population/sample size	Key genes/findings	Main contribution
Harvie et al (2004)^18^	Family Aggregation Study	129 siblings of 64 patients with RCT	-	Siblings had >2× risk for RCT and ∼5× risk for symptoms
Gwilym et al (2009)^16^	Prospective Cohort	Patients with family history of RCT	-	Family history predicted tear development and progression
Tashjian et al (2009)^42^	Genealogical Study (Utah)	Population-level pedigree analysis	-	Demonstrated familial clustering, esp. early-onset cases
Longo et al (2019)^33^	Systematic Review (Candidate Genes)	Review of genetic association studies	*DEFB1*, *FGFR1*, *FGFR3*, *ESRRB*, *FGF10*, *FGF3*	Highlighted potential polymorphisms linked to tendon biology
Teerlink et al (2015)^45^	Genetic Association	176 RCT patients vs. controls	*ESRRB*	Identified 2 high-risk ESRRB haplotypes (OR ∼6.9, ∼5.3)
Tashjian et al (2016)^43^	GWAS	1606 RCT cases + 1206 controls	*SAP30BP*, *SASH1*	First GWAS; implicated apoptosis and cell cycle genes
Kim et al (2021)^24^	GWAS + meta-analysis	21,000+ (UK Biobank + Kaiser)	*SLC39A8*, *UBE2D3*, *STK24*, *FRMPD4*, *ACOT9*, *SAT1*	Identified 11 genome-wide loci; diverse biological pathways
Kim et al (2021)^24^	Transcriptomics	Torn vs. intact RCT tissue	↑*STK24*, ↓*SAT1*, ↓*UBE2D3*	Linked SNPs to gene expression and tendon pathology
Azzarà et al (2022)^2^	Whole-Exome Sequencing (Family)	2 affected, 2 unaffected family members	*COL23A1*, *EMILIN3*, *HDAC10*	Identified rare variants possibly linked to familial RCT

*RCT*, rotator cuff tear; *GWAS*, genome-wide association study; *SNPs*, single nucleotide polymorphisms.

Taken together, current evidence indicates that rotator cuff disease is not solely the result of mechanical degeneration but also reflects inherited susceptibility interacting with biologic processes of tendon degeneration and repair. Family-based studies support a heritable component, and large-scale GWASs provide the strongest population-level evidence identifying specific risk loci. In contrast, transcriptomic and microRNA analyses primarily characterize molecular activity within already diseased tissue rather than primary genetic predisposition.^2^^,^^33^ Although genetic testing is not yet part of routine orthopedic care, understanding these complementary layers of evidence helps explain clinical variability and may inform future risk stratification and biologically targeted treatment strategies in rotator cuff pathology.

## Adhesive capsulitis

Adhesive capsulitis, commonly known as frozen shoulder, is a painful and disabling condition characterized by progressive stiffness and restricted passive range of motion of the glenohumeral joint.^20^ Its pathogenesis has long been debated, with proposed mechanisms involving chronic inflammation, capsular fibrosis, and fibroblastic proliferation.^20^^,^^27^ Although diabetes mellitus and thyroid dysfunction are known risk factors, recent genetic and molecular evidence suggests that hereditary and immunogenetic components also contribute to disease susceptibility.^8^^,^^27^^,^^38^^,^^40^ Genetic evidence in adhesive capsulitis arises from both population-level studies assessing inherited susceptibility and tissue-level investigations characterizing molecular changes within established disease. Distinguishing between these complementary but distinct lines of evidence is essential for appropriate interpretation.

One of the largest studies to date is the GWAS conducted by Green et al, using data from over 10,000 adhesive capsulitis cases in the UK Biobank and replication in the FinnGen cohort.^15^ This study identified 5 genome-wide significant loci associated with adhesive capsulitis, the most notable being the *WNT7B* gene, a known mediator in fibrotic and musculoskeletal pathways.^15^ Interestingly, 3 of the associated loci also showed strong overlap with genetic variants implicated in Dupuytren's disease, another fibroproliferative disorder, supporting the hypothesis of shared pathogenic mechanisms.^15^ The authors also used Mendelian randomization analysis and demonstrated relation between type 1 diabetes and adhesive capsulitis, showing that hyperglycemia may drive disease through glycemic-induced fibrotic signaling rather than mechanical overload.^15^

Supporting this, Kulm et al also conducted a GWAS focused on adhesive capsulitis, identifying significant genetic risk factors beyond environmental or metabolic contributors.^26^ Their analysis supports the idea that genetic liability is a component of the disease, independent from comorbidities such as diabetes and thyroid dysfunction.^26^

In contrast to studies evaluating inherited risk, several investigations have focused on gene expression within affected capsular tissue, providing information on the molecular phenotype of established disease.^36^^,^^37^ Nishimoto et al analyzed synovial tissue from patients undergoing arthroscopic capsular release for adhesive capsulitis and found elevated expression of fibrosis-related genes like *COL1A1*, *COL2A1*, transforming growth factor beta 1 (*TGFB1*), and *SOX9*, as well as matrix metalloproteinases *MMP3*, *MMP9*, and *MMP13*.^36^ These changes were particularly prominent in the axillary recess and rotator interval, supporting a region-specific pathogenic response.^36^ Notably, nerve growth factor (*NGF*) and its receptor (*NGFR*), known to mediate pain and fibrosis, were upregulated in affected tissues, providing a molecular explanation for the chronic pain and restricted motion observed clinically.^36^ Although these findings help explaining the molecular pathology of adhesive capsulitis, they represent acquired changes in gene expression within diseased tissue rather than inherited genetic susceptibility. Such fibrosis and inflammation-related transcriptional changes likely reflect the local biological response to injury or chronic inflammation, not genetic polymorphisms.

The genetic foundation of inflammation in adhesive capsulitis has been shown in multiple Mendelian randomization studies. Li et al identified associations between elevated systemic levels of GROα (growth-regulated oncogene alpha), IP-10 (interferon gamma-induced protein 10), and CCL5 (RANTES) with increased adhesive capsulitis risk.^30^ These cytokines are known mediators of immune cell recruitment and fibrotic transformation. Interestingly, no association was found between adhesive capsulitis and reverse elevation of these cytokines, suggesting that inflammation precedes clinical disease rather than results from it.^30^ Similarly, Ouyang et al confirmed connections between IP-10 and RANTES and adhesive capsulitis, while also demonstrating protective effects of higher systemic levels of tumor necrosis factor-alpha, interleukin - 5, and stromal cell-derived factor-a; showing complex dynamics that influence susceptibility and progression.^37^

Genetic variation in ECM regulation has also been implicated.^48^ Xu et al performed a case-control study in a Chinese Han population and demonstrated that polymorphism in the *MMP3* gene, a key enzyme involved in matrix remodeling, was significantly associated with increased risk of idiopathic frozen shoulder.^48^ Given the known fibrotic and contractile changes in adhesive capsulitis, dysregulation of MMP pathways is a biologically plausible and clinically relevant finding.^48^ Additionally, a systematic review and meta-analysis by Prodromidis et al synthesized data from 7 studies, including twin, familial, and immunogenetic research, and concluded that adhesive capsulitis shows moderate heritability, with an estimated genetic contribution of approximately 42%.^40^ The review also noted elevated prevalence of HLA-B27 in patients with adhesive capsulitis and a higher incidence among individuals of European ancestry, further supporting the genetic component in disease pathogenesis (Table III).^40^

**Table III:** Summary of genetic and molecular studies on adhesive capsulitis.

Study	Study type	Key findings	Implications
Green et al (2021)^15^	GWAS (UK Biobank + FinnGen)	Identified 5 genome-wide loci, especially *WNT7B*; overlap with Dupuytren's disease; showed type 1 diabetes as a causal factor.	Shared fibrotic pathways; role of diabetes in disease pathogenesis.
Kulm et al (2022)^26^	GWAS (independent analysis)	Demonstrated significant heritability and genetic risk beyond metabolic/environmental factors.	Confirmed independent genetic component of disease.
Nishimoto et al (2022)^36^	Gene expression (capsular tissue)	Upregulation of *COL1A1*, *TGFB1*, MMPs, *SOX9*, *NGF*/*NGFR* in axillary recess and rotator interval.	Supports region-specific fibrosis and pain; potential molecular targets.
Li et al (2024)^30^	Mendelian randomization	Causal association between cytokines (GROα, IP-10, CCL5) and disease onset.	Suggests immune dysregulation precedes clinical symptoms.
Ouyang et al (2024)^37^	Mendelian randomization	Causal role of IP-10, RANTES; protective effect of TNF-α, IL-5, SDF-1α.	Highlights complex immune regulatory network.
Xu et al (2016)^48^	Case-control (Chinese Han)	Polymorphism in *MMP3* gene associated with higher risk.	Connects ECM remodeling genes with disease susceptibility.
Prodromidis et al (2016)^40^	Systematic review and meta-analysis	Heritability estimated at ∼42%; higher HLA-B27 prevalence; higher incidence in European ancestry.	Strong evidence of genetic predisposition.

*ECM*, extracellular matrix; *GWAS*, genome-wide association study; *IL-5,* interleukin-5; *SDF-1a,* stromal cell-derived factor-1a; *TNF-a,* tumor necrosis factor-alpha.

Taken together, population-based genetic studies and Mendelian randomization analyses support a measurable inherited susceptibility to adhesive capsulitis, particularly involving immune and fibrotic pathways. In contrast, tissue-level gene expression studies primarily characterize molecular processes active within established disease and help explain the capsular fibrosis and pain observed clinically rather than primary genetic predisposition. For orthopedic surgeons, current genetic evidence improves understanding of why certain patients may be biologically predisposed to frozen shoulder, while molecular findings clarify the pathologic processes encountered at the time of intervention. Although these findings do not yet alter routine surgical management, they provide a more structured approach for interpreting disease variability and may inform future approaches to risk stratification and targeted therapies.

## Glenohumeral osteoarthritis

GHOA is a progressive degenerative joint disorder that results in pain, stiffness, and functional impairment of the shoulder.^5^^,^^10^ Despite being a common indication for shoulder arthroplasty in older adults, the molecular and genetic mechanisms underlying GHOA remain poorly understood compared to weight-bearing joints such as the knee or hip.^10^ Most available shoulder-specific data derive from small transcriptomic studies of surgical specimens rather than large population-based genetic analyses.

In a recent work by Casagrande et al articular cartilage samples from 16 osteoarthritic humeral heads and 10 nonosteoarthritic controls were analyzed using quantitative real-time polymerase chain reaction to assess the expression of 19 genes associated with ECM remodeling, inflammation, catabolism, and intercellular signaling.^5^ Among the most striking findings was the upregulation of connexin 43 (Cx43), a gap junction protein not previously studied in human shoulder cartilage.^5^ Expression of Cx43 was elevated by 85-fold in osteoarthritic samples and was significantly correlated with multiple other OA-related genes, including collagen types I, II, and X, ADAMTS5, versican, MMP-9, and Cox-2.^5^ These associations suggest that Cx43 may not only be a biomarker of cartilage degradation but could also play a regulatory role in coordinating the expression of other degenerative pathways in shoulder OA.^5^

Additional genes found to be significantly overexpressed in osteoarthritic shoulders encoding ADAMTS5, collagen type I, Cox-2, versican, MMP-3, and tumor necrosis factor-alpha. ADAMTS5, a known aggrecanase, was increased 33-fold and is well-established as a driver of cartilage degradation in lower limb OA.^5^ Its correlation with Cx43 further supports it as a core mediator of matrix breakdown in the shoulder.^5^ Similarly, collagen type I, which is not a normal component of healthy articular cartilage, was elevated 13-fold, reflecting a reparative or fibrocartilaginous response to ongoing matrix damage.^5^ These results are consistent with previous findings in OA-affected joints of the lower extremity, showing similarities in osteoarthritic processes across joint types, despite differing mechanical environments.

Beyond cartilage-specific gene expression, Aleem et al demonstrated that glenoid cartilage from GHOA patients exhibits differential upregulation of inflammation- and OA-associated genes including *CCL3*, *CHST11*, *GPR22*, *PRKAR2B*, and *PTGS2* compared to shoulders with instability, further reinforcing the active inflammatory and matrix remodeling of this disease.^1^ Additionally, Lynskey et al found that patients undergoing shoulder arthroplasty for OA with greater disability (higher Quick Disabilities of Arm, Shoulder, and Hand scores) showed increased expression of genes related to interleukins, chemokines, and complement signaling in periarticular soft tissues, implying a broader tissue-level inflammatory state in more symptomatic individuals.^35^ However, the reasons these inflammation- and matrix-related genes are differentially expressed remain unclear. These patterns may reflect acquired molecular responses to mechanical loading, chronic inflammation, or other environmental factors, rather than underlying susceptibility. Finally, a review by Ibounig et al emphasized the urgent need for more shoulder-specific genetic studies, noting the disproportionately sparse data available for GHOA relative to knee or hip OA, despite a comparable clinical burden (Table IV).^21^

**Table IV:** Summary of genetic and molecular studies on glenohumeral osteoarthritis.

Study	Sample	Method	Key findings	Implications
Casagrande et al (2015)^5^	16 GHOA cartilage, 10 controls	qRT-PCR (19 genes)	Cx43 upregulated 85-fold; correlated with collagen I, II, X, ADAMTS5, MMP-9, versican, Cox-2	Cx43 may coordinate degenerative signaling; potential biomarker and therapeutic target
Aleem et al (2023)^1^	Glenoid cartilage from GHOA vs. instability patients	Transcriptome profiling	Upregulation of *CCL3*, *CHST11*, *GPR22*, *PRKAR2B*, *PTGS2* in GHOA	Reveals distinct inflammatory and OA-related gene expression signature in GHOA
Lynskey et al (2024)^35^	Periarticular soft tissues from shoulder arthroplasty	Gene expression correlation with QuickDASH	Increased expression of interleukin, chemokine, complement genes in more disabled patients	Suggests broader tissue-level inflammation and role in symptom severity
Ibounig et al (2021)^21^	Narrative review	Literature synthesis	Sparse genetic data on shoulder OA; highlighted gaps in understanding	Calls for more targeted genetic studies specific to shoulder OA

GHOA, glenohumeral osteoarthritis; OA, osteoarthritis; *qRT-PCR,* quantitative real time - polymerase chain reaction; *QuickDASH,* Quick Disabilities of Arm, Shoulder, and Hand.

Overall, current shoulder-specific evidence in GHOA is derived predominantly from gene expression studies with relatively small sample sizes, which characterize molecular activity within established disease rather than inherited genetic susceptibility. While these consistently demonstrate upregulation of inflammatory and matrix remodeling pathways, they identify associations rather than causal mechanisms. At present, direct genetic risk loci specific to GHOA remain largely undefined, particularly when compared to hip and knee OA.

For orthopedic surgeons, these findings primarily improve understanding of the biologic processes active in advanced disease rather than informing patient-level genetic risk or clinical decision-making. Continued large-scale, shoulder-specific research will be necessary before clinically actionable genetic markers can be established.

## Shoulder instability

Shoulder instability, particularly traumatic anterior instability, is a common problem in young and active individuals and often results in recurrent dislocations and progressive capsular laxity.^25^ Although biomechanical and structural causes have been extensively studied, including bone loss and capsular defects, emerging research has highlighted the role of genetic predisposition in the development and recurrence of shoulder instability.^3^^,^^4^ Familial case reports, population-based studies, and tissue-level gene expression analyses suggest that variations in genes related to collagen synthesis, ECM remodeling, and inflammatory signaling may contribute to capsular weakness and altered healing responses following trauma.^3^^,^^7^ Evidence in shoulder instability derives from both genetic association studies assessing inherited predisposition and tissue-level gene expression analyses reflecting molecular remodeling after injury. Distinguishing between these complementary but distinct mechanisms is essential when interpreting current findings.

Initial clues to a genetic contribution came from case reports of familial aggregation, such as those described by Foëx, where recurrent shoulder dislocations were observed across 3 generations.^13^ A study by Khoschnau et al investigated a polymorphism (rs1800012) in the *COL1A1* gene and found that individuals homozygous for this polymorphism had a significantly lower risk of shoulder dislocations and cruciate ligament injuries in a Swedish cohort, indicating enhanced connective tissue strength due to altered collagen transcription.^23^ This protective association was supported in subsequent work by Collins et al, who reported similar findings in a South African population, further reinforcing the role of *COL1A1* variants in modulating the risk of soft tissue ruptures, including shoulder instability.^9^ Together, these studies highlight the potential for collagen-related genetic polymorphisms to serve as biomarkers of capsular integrity and predisposition to instability.^9^^,^^23^

To further clarify the molecular basis of instability, Belangero et al analyzed gene expression in capsular tissues obtained during surgery for traumatic anterior instability.^4^ They demonstrated that *COL1A1* expression was significantly elevated in affected tissues, and the *COL1A1*/*COL1A2* ratio was markedly increased across multiple regions of the capsule.^4^ Importantly, this altered ratio appeared to diminish with longer symptom duration, suggesting a potential window of altered collagen turnover in early disease stages.^4^ Additionally, *COL1A1*/*COL5A1* imbalance was observed in the anteroinferior and posterior regions, which may explain the regional vulnerability of the capsule in instability-prone shoulders.^4^ These findings raise the possibility that differences in collagen subtype expression may contribute to capsular biomechanical insufficiency, although it remains uncertain whether such changes reflect inherited predisposition or secondary remodeling following trauma. On the other hand, such findings do not establish whether these changes of protein expression merely represent acquired responses to trauma and inflammation. Without preinjury expression levels, it remains unclear whether these collagen imbalances reflect a pre-existing predisposition or a secondary remodeling process following repeated instability episodes.

The role of *TGFB1* and its receptor *TGF* receptor beta 1 has also been implicated in shoulder instability.^3^^,^^4^^,^^7^ Expression of these genes, along with downstream regulators of collagen crosslinking such as *LOX* and *PLOD2*, was found to be altered in patients with instability, suggesting dysregulated fibrotic signaling and remodeling.^3^^,^^4^^,^^7^ Parallel evaluation of glycoproteins revealed increased expression of fibronectin 1 and tenascin C in the anteroinferior capsule, with fibronectin 1 levels correlating with symptom duration and recurrence.^3^^,^^4^^,^^7^ These findings indicate that not only structural collagens but also ECM-modifying proteins contribute to the instability.^7^ As with collagen-related findings, these expression changes likely reflect active capsular remodeling in response to injury and recurrence rather than direct evidence of inherited susceptibility.

From a methodological standpoint, gene expression analysis in shoulder instability has been refined by identifying optimal reference genes for reverse transcription - quantitative polymerase chain reaction. Leal et al compared 6 candidate genes across different regions of the capsule in both patients and controls and found that *HPRT1* and *B2M* offered the most stable normalization.^28^ Using this approach, they confirmed elevated *COL1A1* expression in the anterosuperior and posterior capsule of instability patients compared to controls, further supporting the notion of regionalized gene regulation in capsular remodeling (Table V).^28^

**Table V:** Summary of genetic and molecular studies on shoulder instability.

Study	Sample	Method	Key findings	Implications
Foëx et al (2001)^13^	Familial case reports (3 generations)	Case series	Documented familial aggregation of recurrent shoulder dislocations	Suggested a possible hereditary predisposition to shoulder instability
Khoschnau et al (2008)^23^	Swedish cohort with shoulder dislocation	Genetic association study (rs1800012)	Homozygosity for *COL1A1* polymorphism conferred protection against shoulder dislocations	Indicates collagen transcription may influence capsular integrity
Collins et al (2010)^9^	South African population	Genetic association study	Confirmed protective effect of COL1A1 variant in a second cohort	Reinforces role of *COL1A1* polymorphisms in soft tissue resilience
Belangero et al (2014)^3^	Capsular tissue from instability patients	RT-qPCR, tissue-level gene expression	Elevated *COL1A1* expression; altered *COL1A1*/*COL1A2* and *COL1A1*/*COL5A1* ratios across capsule regions	Suggests region-specific collagen imbalance contributes to capsular laxity
Leal et al (2014)^28^	Capsular tissue from patients and controls	RT-qPCR normalization study	Identified *HPRT1* and *B2M* as optimal reference genes; confirmed upregulated *COL1A1* in instability samples	Supports robust gene expression profiling in regional capsular analysis
Belangero et al (2016)^4^	Same cohort, extended analysis	Gene expression of *TGFB1*, *TGFBR1*, *LOX*, *PLOD2*, *FN1*, *TNC*	Altered fibrotic and ECM signaling in anteroinferior/posterior capsule; *LOX* downregulated in chronic cases	Highlights temporal and regional variation in remodeling; fibrotic dysregulation as a factor in recurrence
Belangero et al (2016)^4^	Additional genes in instability cohort	Gene expression (*COMP*, *FN1*)	Reduced *COMP* and increased *FN1* in inflamed capsule; *FN1* correlated with symptom duration	Indicates failed matrix repair and reduced capsular resilience in chronic instability

FN1, fibronectin 1; *RT-qPCR,* reverse transcription - quantitative polymerase chain reaction; TGFB1, transforming growth factor beta 1; TGFBR1, transforming growth factor receptor beta 1; TNC, tenascin C.

Accordingly, while most transcriptomic studies focus on local capsular remodeling after injury, clinicians must also remain attentive to the possibility of an underlying systemic connective tissue predisposition in selected patients. If shoulder instability is recurrent, and the patient demonstrates additional features suggestive of a connective tissue disorder such as Ehlers-Danlos syndrome (eg, skin hyperextensibility, easy bruising, atrophic scarring, or generalized joint hypermobility) or reports a family history of similar symptoms, referral to medical genetics for germline evaluation may be considered.

Overall, limited but consistent evidence suggests that inherited collagen-related polymorphisms may influence susceptibility to shoulder instability. In contrast, most available transcriptomic data characterize molecular changes within the capsule after injury and recurrent dislocation, reflecting adaptive or maladaptive remodeling rather than primary genetic risk. For orthopedic surgeons, current genetic insights help explain why certain individuals may demonstrate greater capsular laxity or recurrence risk, but they do not yet support routine genetic testing or alter established surgical decision-making. Further longitudinal and population-based studies will be necessary to clarify the relative contributions of inherited predisposition and post-traumatic molecular remodeling.

## Conclusion

Current evidence demonstrates that several common shoulder pathologies, RCTs and adhesive capsulitis, have measurable inherited components supported by family-based GWAS. In contrast, much of the available data on GHOA and shoulder instability derive from transcriptomic analyses of diseased tissue, which characterize molecular remodeling rather than primary genetic susceptibility. These findings collectively suggest that genetic predisposition and biologic processes both contribute to disease expression, although the strength and type of evidence vary by condition.

At present, genetic insights primarily enhance understanding of disease heterogeneity rather than directly influence surgical decision-making. For orthopedic surgeons, familiarity with the genetic basis of shoulder disease supports interpretation of emerging research and may, with further investigation, help inform patient selection and prognosis in the evolving era of precision medicine.

## Disclaimers:

Funding: No funding was disclosed by the authors.

Conflicts of interest: The authors, their immediate families, and any research foundations with which they are affiliated have not received any financial payments or other benefits from any commercial entity related to the subject of this article.
